# Subjective Values in Decision-Making Under Uncertainty: A Systematic Review

**DOI:** 10.3390/bs16091494

**Published:** 2026-08-26

**Authors:** Meihui Zheng, Zhenzhong Ma, Yiru Li, Dapeng Liang

**Affiliations:** 1 School of Business, Harbin Institute of Technology, Harbin 150001, China; 15764231153@163.com; 2 School of Economics and Management, Harbin Institute of Technology Shenzhen, Shenzhen 518055, China; 3 Odette School of Business, University of Windsor, Windsor, ON N9B 3P4, Canada; 4 School of Management, Harbin Institute of Technology, Harbin 150001, China; liyiruuu@gmail.com (Y.L.); ldp0920@hit.edu.cn (D.L.)

**Keywords:** subjective value, neuroeconomics, uncertainty decision-making, concept evolution, theoretical foundations, measurement, VOSviewer

## Abstract

Decision-making under uncertainty, risk, and ambiguity is a central topic in behavioral sciences, yet the cognitive and neural mechanisms through which individuals construct subjective value remain fragmented across disciplines. Neuroeconomics has emerged as an interdisciplinary field integrating psychology, economics, and neuroscience to explain how subjective value guides judgment and choice under uncertain conditions. However, the conceptual foundations of subjective value and its role across different forms of uncertainty remain insufficiently integrated. This study provides the first comprehensive review of subjective value research by combining a systematic literature review with bibliometric analysis. We first trace the theoretical evolution of judgment and decision-making under uncertainty, emphasizing the development of subjective value as a core construct linking cognitive evaluation, affective processing, and behavioral choice. We then review major approaches to measuring subjective value and examine their contributions to understanding individual differences in decision-making. Using VOSviewer, we identify the intellectual structure, major research themes, and emerging trends in the field. The findings reveal two complementary knowledge foundations: theories of judgment and decision-making under uncertainty and the neural mechanisms underlying subjective value representation. Three dominant research themes emerge: (1) cognitive and affective processes underlying subjective value construction during uncertainty-related judgment; (2) neural representations of subjective value across risky and ambiguous decision contexts; and (3) the development of integrative cross-domain models that connect psychological, economic, and neuroscientific perspectives on decision-making. Based on these findings, we propose future research directions that emphasize distinguishing risk, ambiguity, and uncertainty at both behavioral and neural levels, integrating contextual and individual-difference factors into subjective value models, and extending neuroeconomic research beyond laboratory paradigms to real-world decision environments. By synthesizing theoretical and empirical advances across disciplines, this review offers an integrative framework for understanding how subjective value shapes human judgment and decision-making under uncertainty.

## 1. Introduction

Judgment and decision-making are fundamental cognitive processes through which individuals evaluate alternatives and select courses of action (Simon, 1986). Although decisions may occur under conditions of certainty, most real-world decisions—ranging from financial investment to consumer behavior, education, and organizational strategy—involve varying degrees of uncertainty (Edelson et al., 2018). Knight’s (1921) seminal distinction between risk, in which outcome probabilities are known, and ambiguity, in which probabilities are unknown or difficult to specify, established the conceptual foundation for contemporary research on decision-making under uncertainty. This distinction has since become central to behavioral economics, psychology, and neuroscience, providing a framework for understanding how people make judgments when objective information is incomplete.

Research on judgment and decision-making under uncertainty has traditionally focused on observable choice behavior and decision outcomes. Extensive studies have examined ambiguity aversion and its psychological determinants (FeldmanHall et al., 2016), individual differences in ambiguity attitudes (Dimmock et al., 2016; Trautmann & Van de Kuilen, 2015), the stability of ambiguity preferences across contexts (Eliaz & Ortoleva, 2016; Li et al., 2018), the relationships between decisions under risk and ambiguity (Golman et al., 2021; Lauriola et al., 2007), applications of ambiguity in real-world decision environments (Holm et al., 2013; Julio & Yook, 2016), and the comparative performance of competing behavioral decision models (Hey et al., 2010; Williams et al., 2021). While these studies have substantially advanced our understanding of behavioral responses to uncertainty, they infer underlying psychological processes mainly from observed choices and therefore provide limited insight into how individuals internally construct value before making a decision.

Recent developments in neuroeconomics have shifted attention from decision outcomes to the cognitive and neural mechanisms that generate judgments under uncertainty. Integrating psychology, economics, and neuroscience, neuroeconomics seeks to explain how subjective preferences are represented in the brain and translated into behavior. Central to this framework is the concept of subjective value—the internal valuation individuals assign to available options by integrating objective information with cognitive evaluation, affective responses, prior experience, and contextual influences. Contemporary decision process models consistently identify value computation as a prerequisite for choice. For example, Rangel et al. (2008) conceptualized decision-making as a sequence comprising representation, valuation, action selection, outcome evaluation, and learning, whereas Glimcher (2011) argued that valuation precedes and guides choice. These frameworks indicate that subjective value constitutes an intermediate cognitive representation rather than a direct reflection of observable decisions. Consistent with this distinction, empirical evidence has demonstrated that subjective value and decision outcomes are related but not interchangeable, with nonlinear relationships often emerging under conditions of uncertainty (FeldmanHall et al., 2016).

The growing emphasis on subjective value has substantially expanded the scope of uncertainty research. Rather than treating choices as the primary object of analysis, neuroeconomics investigates the cognitive and neural computations through which people evaluate uncertain alternatives before acting. As Fehr and Rangel (2011) argued, this research develops along two complementary lines. Behavioral studies estimate subjective value from observed choices using computational models grounded in decision theory, whereas neuroscientific studies employ techniques such as functional magnetic resonance imaging (fMRI) to identify neural representations of subjective value. Together, these approaches provide converging evidence for understanding the mechanisms underlying judgment and decision-making under uncertainty.

Despite rapid progress, two important challenges remain. First, although subjective value has become the cornerstone of decision-making, its conceptual boundaries remain insufficiently defined. Different theoretical traditions conceptualize subjective value differently, and its evolution across research on risk, ambiguity, and broader uncertainty has not been systematically synthesized. This lack of conceptual integration limits cumulative theory development and complicates comparisons across behavioral and neuroscientific studies (Truc, 2023). Second, despite the rapid expansion of neuroeconomic research, there is still no comprehensive review devoted specifically to subjective value in decision-making under uncertainty. Existing reviews either provide broad overviews of neuroeconomics or discuss subjective value very briefly (Glimcher, 2011; Levy, 2017; Padoa-Schioppa, 2022; Zhu & Wang, 2015), leaving the intellectual structure, major research themes, and future directions of this rapidly developing field largely unexplored.

To address these gaps, this review combines a systematic literature review with bibliometric analysis using VOSviewer (version 1.6.20) to provide an integrated assessment of subjective value research in judgment and decision-making under uncertainty. Specifically, we pursue two objectives. First, we synthesize the conceptual evolution of subjective value by examining its theoretical foundations, its development across decision-making under risk and ambiguity, and contemporary behavioral and neural approaches to its measurement. Second, we map the intellectual landscape of the field by identifying its research foundations, leading contributors, dominant themes, and emerging research directions. By integrating evidence from psychology, economics, and neuroscience, this review provides a comprehensive framework for understanding how subjective value shapes human judgment and decision-making under conditions of risk, ambiguity, and uncertainty, while identifying promising directions for future interdisciplinary research.

## 2. Connotation, Theoretical Basis, and Measurement

### 2.1. Conceptualization of Subjective Value

#### 2.1.1. Subjective Value Across Disciplinary Perspectives

Subjective value refers to an individual’s internal evaluation of the desirability or worth of an object, outcome, or decision option (Kahneman & Tversky, 1979; Von Neumann & Morgenstern, 1944). Rather than reflecting objective value, subjective value captures how people integrate available information with their preferences, expectations, and psychological states when making judgments and choices (Kahneman & Tversky, 1979).

The concept of subjective value has been extensively discussed in Austrian economics, behavioral economics, and neuroeconomics, with each emphasizing different aspects of valuation. The Austrian school views value as inherently subjective and rooted in individual preferences. As individuals differ in their needs, experiences, and goals, they assign different values to the same object or decision alternative. Accordingly, Austrian economists reject the assumption that value can be objectively determined or universally shared (Menger, 2007). However, subjective valuation is not unique to Austrian economics. Neoclassical expected utility theory also incorporates subjective valuation by representing an individual’s preferences through a utility function. Under this framework, individuals are assumed to evaluate alternatives according to their own preferences while making rational choices that satisfy consistency axioms and maximize expected utility. Thus, valuation is subjective in its dependence on individual preferences but rational and internally consistent in the neoclassical framework. In this respect, both Austrian and neoclassical traditions recognize the individual-specific nature of value, although they differ substantially in their theoretical foundations and treatment of choice. Moreover, the Austrian school does not generally characterize individual valuation in terms of the systematic cognitive biases or predictable deviations from rational choice that later became central to behavioral economics (Menger, 2007). This view is different from the understanding of subjective value in behavioral economics, as discussed below.

Behavioral economics extends research on subjective value by recognizing that subjective valuation is systematically influenced by cognitive limitations and psychological biases. Rather than assuming fully rational decision-makers, behavioral economists demonstrate that judgments frequently deviate from normative models because of heuristics, framing effects, and reference dependence (Kahneman & Tversky, 1979). Nevertheless, subjective value remains fundamentally individual-specific, reflecting each person’s unique psychological evaluation of uncertain outcomes (Peysakhovich & Karmarkar, 2016).

Neuroeconomics further advances the concept by investigating the neural mechanisms through which subjective value is represented and computed. Rather than emphasizing whether decisions are rational or irrational, neuroeconomics conceptualizes subjective value as a common neural currency that enables the comparison of qualitatively different alternatives. Neural representations of subjective value have been identified across a wide range of behaviors, including decision-making under uncertainty, intertemporal choice, effort allocation, information search, and prosocial behavior (Kobayashi & Hsu, 2019; Schultz et al., 2015). From this perspective, diverse experiences and rewards are translated into a common value representation that supports judgment and choice.

These perspectives converge on the view that subjective value is an internal, individual-specific evaluation that guides decision-making. However, they differ in their theoretical emphasis; in particular, Austrian economics focuses on individual preference, behavioral economics highlights cognitive and psychological influences on valuation, and neuroeconomics seeks to uncover the neural computations underlying subjective value.

#### 2.1.2. Subjective Value Across Different Levels of Decision-Making Under Uncertainty

In research on judgment and decision-making under uncertainty, the term subjective value is used at different, but computationally related, levels of the valuation process. To avoid implying that these levels constitute logically independent forms of value, we distinguish between outcome-level valuation, option-level integrated valuation, and choice-level comparative valuation. These levels are hierarchical: the subjective values assigned to potential outcomes provide inputs to the integrated valuation of an option, and the resulting option values provide the basis for comparison and choice. Thus, the distinction is analytical rather than one of logical independence.

The first level, outcome-level subjective value, concerns the psychological valuation of a potential consequence, such as a particular monetary gain or loss. In prospect theory, for example, the value function transforms an objective outcome, defined relative to a reference point, into its subjective psychological value (Kahneman & Tversky, 1979; Tversky & Kahneman, 1992). This quantity captures how desirable or undesirable a potential outcome is to the decision-maker but does not by itself represent the overall subjective value of an uncertain option. Decisions under uncertainty additionally require consideration of the likelihood associated with each possible outcome (Glimcher, 2011). Outcome-level valuation should therefore be understood as a constituent component of, rather than an alternative to, option-level valuation.

The second level, option-level integrated subjective value, combines outcome valuations with assessments or transformations of their associated probabilities. For example, in expected utility theory, the value of an uncertain option can be represented by the probability-weighted utilities of its possible outcomes, whereas in prospect theory, it is determined by integrating the subjective values of outcomes with decision weights derived from probabilities (Kahneman & Tversky, 1979; Tversky & Kahneman, 1992). Accordingly, the distinction between outcome value and option value does not imply that the former “stands alone” logically from probability assessment. Rather, outcome-level subjective value is one computational input into the integrated subjective valuation of the option. This integrated quantity represents the overall attractiveness of a risky or ambiguous option and is frequently termed subjective value or expected subjective value in behavioral and neuroeconomic research (Edelson et al., 2018; FeldmanHall et al., 2016; Wang et al., 2019; Williams et al., 2021).

The third level, choice-level comparative subjective value, concerns the relative valuation that directly informs choice between competing options. Once integrated subjective values have been computed for individual alternatives, decision-makers compare them, and choice can be modeled as a function of their value difference. In the widely used binary choice paradigm, this quantity can be expressed as the expected subjective value of one option minus that of the alternative and is therefore referred to here as the expected subjective value difference (Birnbaum & Bahra, 2007; Peysakhovich & Karmarkar, 2016; Wu & Markle, 2008). Thus, this third level is also not an independent form of valuation; rather, it represents a comparative transformation of option-level subjective values that links valuation to observed choice.

This hierarchical distinction is particularly important when interpreting binary choice paradigms, which commonly involve gain-only, loss-only, or mixed gain–loss decisions. In some mixed paradigms, one alternative is operationalized as a reference or safe option with zero potential gain and zero potential loss, whereas the competing alternative involves risky or ambiguous outcomes. When the subjective value assigned to the reference option is normalized to zero, the subjective value difference between the two alternatives becomes numerically equivalent to the integrated subjective value of the risky or ambiguous option. Consequently, studies employing such paradigms may use subjective value to describe what is computationally both an option value and, relative to the zero-valued reference alternative, a value difference (Edelson et al., 2018). By contrast, in gain-only or loss-only paradigms in which both alternatives have non-zero subjective values, the distinction becomes consequential: the value of either option alone does not represent the comparative signal underlying choice, which is more precisely characterized by the subjective value difference between the alternatives.

Accordingly, the three levels identified here should not be interpreted as three separate or competing conceptions of subjective value. Instead, they describe successive components of a common valuation-to-choice process: potential outcomes are subjectively valued; these outcome values are integrated with probability information to generate option-level subjective values; and option values are compared to generate a relative value signal that guides choice. The same term subjective value has nevertheless been used across the literature for quantities at different points in this process, creating potential conceptual ambiguity. Explicitly distinguishing outcome-level, option-level, and choice-level valuation therefore provides a more precise framework for comparing behavioral and neuroeconomic studies and for interpreting the neural signals associated with valuation and decision-making under uncertainty (see Table 1 for more information).

### 2.2. Evolution of the Concept of Subjective Value and Theoretical Foundations of Decision-Making Under Uncertainty

**Figure 1:**
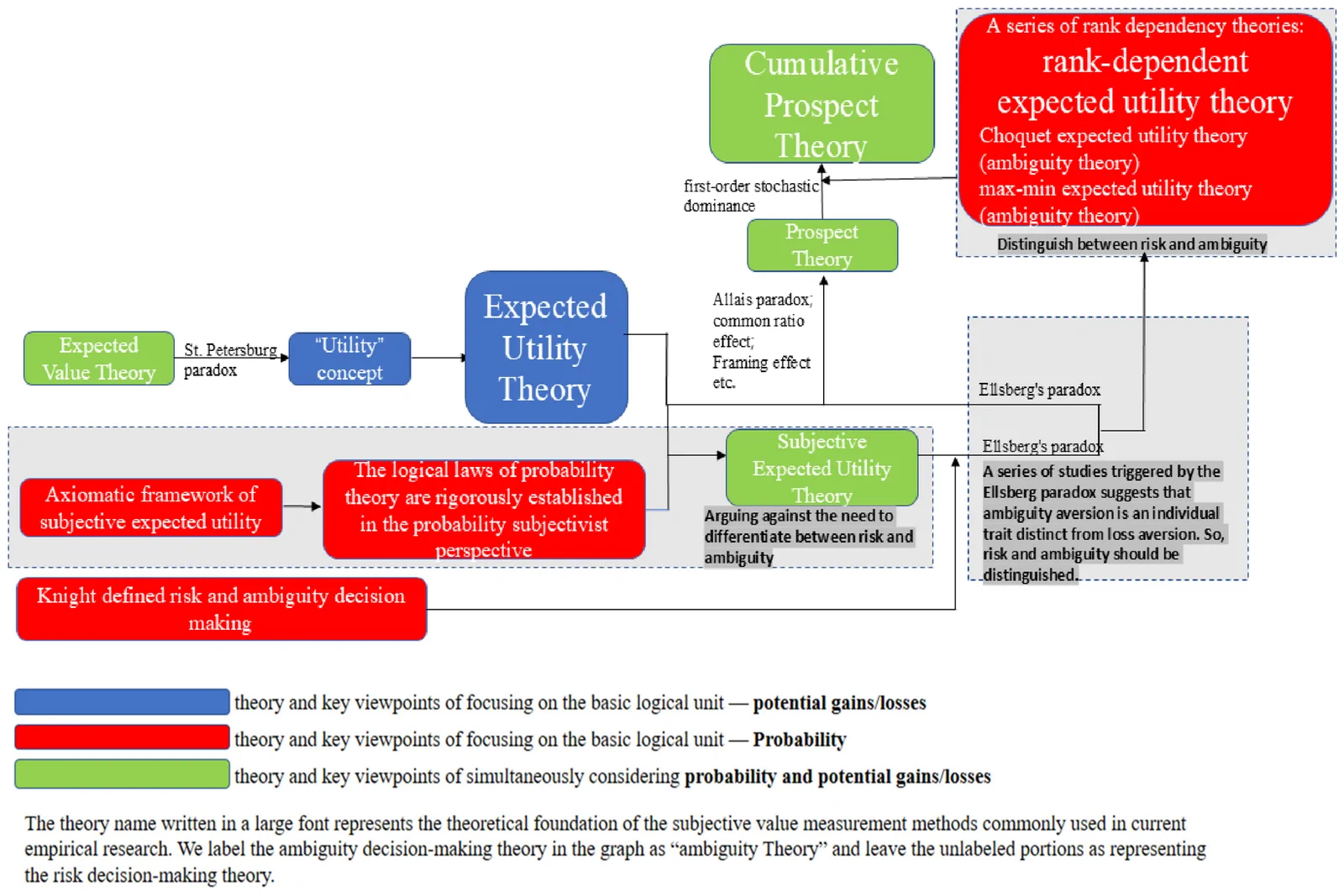
Theoretical foundations of subjective value in decision-making under uncertainty.

The meaning of subjective value has evolved alongside theories of decision-making under uncertainty. This development reflects two fundamental questions: how individuals evaluate potential gains and losses and how they judge the likelihood of uncertain outcomes. Over time, theories have moved from objective representations of outcomes and probabilities toward increasingly psychological accounts in which both valuation and probability judgment are subjective. More recently, neuroeconomics has further reconceptualized subjective value as an internal neural representation involved in choice. Table 2 summarizes this theoretical evolution, while Figure 1 illustrates the relationships among the major theories.

#### 2.2.1. From Expected Value to Subjective Utility

Expected value theory provided the earliest formal framework for choice under uncertainty. Pascal conceptualized uncertain options as combinations of potential outcomes and their probabilities, with expected value calculated from objective outcomes and objective probabilities (Pascal, 1670, 1966; Todhunter, 1865; Glimcher, 2011). This framework established outcome magnitude and probability as the two basic components of uncertain choice.

The limitations of this normative perspective became apparent with the St. Petersburg paradox, which demonstrated that people’s preferences often diverge systematically from expected value maximization. Bernoulli (1738, 1954) therefore introduced the concept of utility, arguing that individuals respond to the subjective satisfaction generated by outcomes rather than to their objective monetary value. Building on this insight, Von Neumann and Morgenstern (1944) developed expected utility theory, which retained objective probabilities but replaced objective outcome values with subjective utility. This shift established the first major foundation of subjective value: the value of an outcome depends on the decision-maker’s psychological evaluation rather than on its objective magnitude alone.

#### 2.2.2. From Objective to Subjective Probability

A second theoretical development concerned the nature of probability judgment. Bernoulli (1954) regarded probability partly as a degree of belief, whereas Von Neumann and Morgenstern (1944) treated probabilities as objective quantities derived from repeated observations. In many real-world settings, however, objective probabilities are unavailable or impossible to estimate reliably (Xu et al., 2008).

Knight (1921) formalized this distinction by separating risk, where probabilities are known, from ambiguity, where probabilities cannot be specified objectively. Ramsey (1931) and Finetti (1931), however, challenged this sharp distinction by arguing that individuals can assign subjective probabilities to uncertain events. Their work established subjective probability as a measurable representation of belief and laid the foundation for Bayesian approaches to uncertainty.

Savage’s (1954) subjective expected utility theory integrated these two developments by treating both utility and probability as subjective. Objective probabilities were regarded as a special case of subjective beliefs, thereby reducing the theoretical distinction between risk and ambiguity. This framework substantially broadened the meaning of subjective value by recognizing that both outcome evaluation and probability judgment can reflect individual beliefs.

#### 2.2.3. Re-Establishing the Distinction Between Risk and Ambiguity

The assumption that risk and ambiguity can be treated within a unified subjective probability framework was challenged by several behavioral paradoxes. Expected utility theory could not adequately explain the Allais paradox, framing effects, the common-ratio effect, or the Ellsberg paradox (Allais, 1953; Ellsberg, 1961; Kahneman & Tversky, 1979; MacCrimmon & Larsson, 1979). Similarly, subjective expected utility theory struggled to explain systematic ambiguity aversion (Ellsberg, 1961; Slovic & Tversky, 1974).

The Ellsberg paradox was particularly influential because it demonstrated that individuals respond differently to known and unknown probabilities, often preferring risky options over ambiguous ones even when expected outcomes are comparable (Ellsberg, 1961). Subsequent evidence showed that ambiguity attitudes and risk attitudes are not interchangeable and that people may persist in ambiguity-averse choices even after recognizing their logical inconsistency (Cohen et al., 1985; MacCrimmon & Larsson, 1979; Slovic & Tversky, 1974). These findings restored the theoretical significance of Knight’s (1921) distinction and suggested that subjective value may be constructed differently under risk and ambiguity.

#### 2.2.4. Prospect Theory and the Behavioral Reconstruction of Subjective Value

Following these challenges to normative theories, Kahneman and Tversky (1979) proposed prospect theory, which reconceptualized subjective value from a behavioral perspective. Rather than evaluating final wealth, individuals assess gains and losses relative to a reference point. The resulting value function captures diminishing sensitivity and asymmetry between gains and losses.

Prospect theory also transformed probability judgment. Instead of assuming that objective probabilities enter valuation directly, it proposed probability weighting, whereby low probabilities tend to be overweighted and high probabilities underweighted (Glimcher, 2011; Kahneman & Tversky, 1979). Subjective value therefore became the combined product of psychologically transformed outcomes and psychologically weighted probabilities.

Further theoretical development distinguished probability weights from decision weights. Rank-dependent expected utility theory proposed that the weight assigned to an outcome depends not only on its own probability but also on its rank relative to other outcomes (Quiggin, 1982). This insight, combined with prospect theory, contributed to the development of cumulative prospect theory (Tversky & Kahneman, 1992), which introduced separate weighting functions for gains and losses and became a major behavioral foundation for estimating subjective value under risk.

Ambiguity research developed along a partly separate trajectory. Gilboa and Schmeidler (1989) proposed the Maxmin Expected Utility Model, while Schmeidler (1989) introduced Choquet Expected Utility to address non-additive beliefs under ambiguity. These models provided important foundations for later attempts to estimate subjective value when probability information is incomplete (FeldmanHall et al., 2016; Levy et al., 2010).

#### 2.2.5. Subjective Value in Neuroeconomics

Neuroeconomics introduced a further conceptual shift by linking behavioral theories of valuation to neural processes. Rather than treating subjective value solely as a latent construct inferred from choice, neuroeconomic research examines how value-related information is represented in neural systems involved in decision-making. Activity in regions such as the medial prefrontal cortex and striatum has therefore been used as a neural correlate of subjective value (Glimcher, 2011).

This development created an interdisciplinary framework linking economic utility, behavioral valuation, and neural representation. Expected utility theory initially provided an important economic foundation, while behavioral models such as cumulative prospect theory were subsequently incorporated to account for systematic deviations from normative choice (Edelson et al., 2018; FeldmanHall et al., 2016; Levy et al., 2010; Williams et al., 2021).

However, this integration also reveals an unresolved theoretical tension. Expected utility theory, prospect theory, and neuroeconomic models are grounded in different assumptions about rationality, probability processing, and the meaning of utility. Treating subjective value as a direct neural counterpart of economic utility may therefore oversimplify these conceptual differences. A central challenge for future neuroeconomic research is to clarify how behavioral constructs derived from competing theories map onto neural representations, particularly when distinguishing risk from ambiguity.

Overall, the evolution of subjective value reflects a broader transition from objective expected outcomes, to subjective utility and probability weighting, and finally to behavioral and neural representations of value. This progression has substantially advanced understanding of judgment under uncertainty, while also revealing important unresolved questions concerning the relationship between risk and ambiguity, the psychological construction of probability, and the correspondence between behavioral and neural measures of subjective value.

### 2.3. Measurement of Subjective Value in Decision-Making Under Uncertainty

#### 2.3.1. Behavioral Measurement Under Risk

In behavioral research, subjective value under risk is typically estimated using cumulative prospect theory or expected utility theory. Researchers first estimate individual-level parameters—such as loss aversion, probability weighting, and risk attitude—using maximum likelihood estimation (Edelson et al., 2018; FeldmanHall et al., 2016; Levy et al., 2010; Williams et al., 2021) or least-squares estimation (Tversky & Kahneman, 1992). These parameters are then used to derive the subjective value assigned to each risky option. This model-based approach provides a systematic way to infer latent valuation processes from observed choices.

#### 2.3.2. Behavioral Measurement Under Ambiguity

Estimating subjective value under ambiguity is more challenging because outcome probabilities are incompletely specified. Existing studies generally follow two approaches. The first combines the value function of cumulative prospect theory with probability adjustments derived from ambiguity models. Representative studies using the occlusion paradigm include Levy et al. (2010) and FeldmanHall et al. (2016). In this paradigm, ambiguity is operationalized primarily as the amount of unknown probability information. Subjective probabilities are then derived by distributing the unknown probability mass and adjusting it using individual ambiguity attitude parameters.

Although widely used, this approach has an important limitation: it focuses on the degree of ambiguity while largely holding constant the valence of known information. In effect, unknown information is often treated as equally likely to produce favorable or unfavorable outcomes (FeldmanHall et al., 2016; Levy et al., 2010). This simplifying assumption may not adequately reflect real-world ambiguous decisions, where known information is often asymmetric and where favorable and unfavorable evidence may influence valuation differently.

The second approach attempts to capture this richer informational structure. Edelson et al. (2018), for example, extended the occlusion paradigm by incorporating the degree of ambiguity together with known favorable and unfavorable information. Their model assumes that individuals first transform ambiguous probabilities into risk-equivalent probabilities and then evaluate the option using principles derived from risky decision-making. This represents an important methodological advance, but the underlying assumption that ambiguity is cognitively converted into risk before valuation remains insufficiently validated.

These approaches therefore reveal a broader measurement challenge: although risk and ambiguity are theoretically distinct, subjective value under ambiguity is still often estimated using models largely inherited from risky decision-making. Developing and comparing ambiguity-specific valuation models remains an important methodological priority.

#### 2.3.3. Neural Measurement and Behavioral–Neural Integration

Neuroeconomic research complements behavioral estimation by examining the neural representation of subjective value. Neural firing rates in valuation-related regions are associated with blood oxygen level-dependent (BOLD) signals measured by functional magnetic resonance imaging (fMRI), making BOLD activity a widely used neural indicator of subjective value (Glimcher, 2011).

A common neuroeconomic strategy is to estimate subjective value from behavioral choice models and then examine whether these estimated values correspond to neural activity measured through fMRI (Edelson et al., 2018; Fehr & Rangel, 2011; FeldmanHall et al., 2016; Levy et al., 2010; Williams et al., 2021). This behavioral–neural convergence strengthens inference by linking observable choices, latent computational parameters, and neural responses.

Each approach nevertheless has limitations. Behavioral estimates are inherently model-dependent, meaning that subjective value can vary with the assumptions and functional forms imposed by the researcher. Neural measures provide valuable spatial information but BOLD signals are indirect and temporally coarse indicators of neuronal activity. Accordingly, behavioral fitting and neural measurement should be treated as complementary rather than interchangeable measures of subjective value. Their careful integration offers the most promising approach for understanding how uncertainty attitudes, loss aversion, probability weighting, and other psychological processes are represented and translated into judgment and choice under risk and ambiguity.

## 3. A VOSviewer-Based Bibliometric Study on Subjective Value Studies

### 3.1. Data Sources and Research Methodology

#### 3.1.1. Data Sources

To provide a comprehensive overview of subjective value research in judgment and decision-making under uncertainty, this review combined a systematic literature search with bibliometric analysis. The literature was retrieved from the Web of Science Core Collection, one of the most widely used databases for bibliometric research because of its broad disciplinary coverage and high-quality indexing. The search covered publications from 1 March 1997, the earliest year in which relevant studies appeared in the database, to 25 December 2022. Following consultation with two experts in neuroeconomics and behavioral decision-making, the final search strategy was as follows: TS = (“subjective value”) AND (ambiguity OR ambiguous OR risk OR risky OR uncertainty). We selected the year 2022 as the endpoint of the bibliometric dataset for the following reasons. Bibliometric analyses based on citation and co-citation patterns require a sufficient citation window for publications to accumulate citations and establish identifiable intellectual relationships. Because the data were collected in 2025, using 2022 as the endpoint provided even the most recent publications in the dataset with approximately three years of citation exposure. Including publications from 2024 or 2025 would systematically disadvantage very recent studies in citation-based analyses and could distort the identification of influential publications, co-citation structures, and established research clusters.

To ensure relevance to the scope of this review, several restrictions were applied. Only English-language journal articles and review papers were included, and the search was limited to four disciplinary categories closely related to subjective value research: management, economics, psychology, and neuroscience. The initial search yielded 1040 publications.

To identify studies specifically addressing subjective value in decision-making under uncertainty, all records were manually screened by examining their titles, abstracts, keywords, research methods, and main contents. Articles that met the following criteria were included in the analysis: (1) articles specifically examining subjective value in decision-making under uncertainty, including both risk and ambiguity; (2) given that many behavioral-level studies operationalize subjective value using the concept of utility, articles employing utility-based measures of subjective value were retained; and (3) studies incorporating mathematical derivations to formulate hypotheses but relying primarily on behavioral or neuroscientific experiments were retained. In addition, articles with the following characteristics were excluded from the analysis: (1) studies mentioning uncertainty, including risk and ambiguity, only as background context rather than as the primary focus of judgment or decision-making; (2) studies devoted primarily to axiomatic or mathematical derivations within neoclassical economic theory, without empirical investigation of subjective value; and (3) articles related to Alzheimer’s disease, not about subjective value per se. After this screening process, 305 publications out of 1040 retrieved records met the selection criteria and formed the final dataset for subsequent analyses.

#### 3.1.2. Research Methodology

This study employed a complementary combination of bibliometric analysis and traditional qualitative literature review to examine the intellectual development of subjective value research (Ma, 2009; Ma et al., 2008, 2012). Bibliometric analysis was conducted using VOSviewer (Van Eck & Waltman, 2010), a visualization software widely used for mapping scientific knowledge because of its robust clustering algorithms and intuitive graphical displays. VOSviewer provides three complementary visualization modes—network, overlay, and density maps—which facilitate the identification of collaboration patterns, intellectual structures, and research trends.

Several bibliometric techniques were employed. Co-authorship analysis was used to identify major research groups and patterns of scientific collaboration. Co-citation analysis was conducted to reveal the foundational literature and theoretical knowledge base of subjective value research. Keyword co-occurrence analysis was used to identify the dominant research themes and conceptual structure of the field. Finally, overlay visualization of keyword co-occurrence was applied to illustrate the temporal evolution of research topics and identify emerging areas of investigation.

The bibliometric findings were complemented by a qualitative synthesis of the full-text literature. Integrating quantitative knowledge mapping with a systematic literature review enabled us to not only describe publication patterns objectively, but also interpret the theoretical evolution, conceptual foundations, and future directions of subjective value research in judgment and decision-making under uncertainty.

### 3.2. Analysis of the Intellectual Landscape

#### 3.2.1. Mapping the Publications

**Figure 2:**
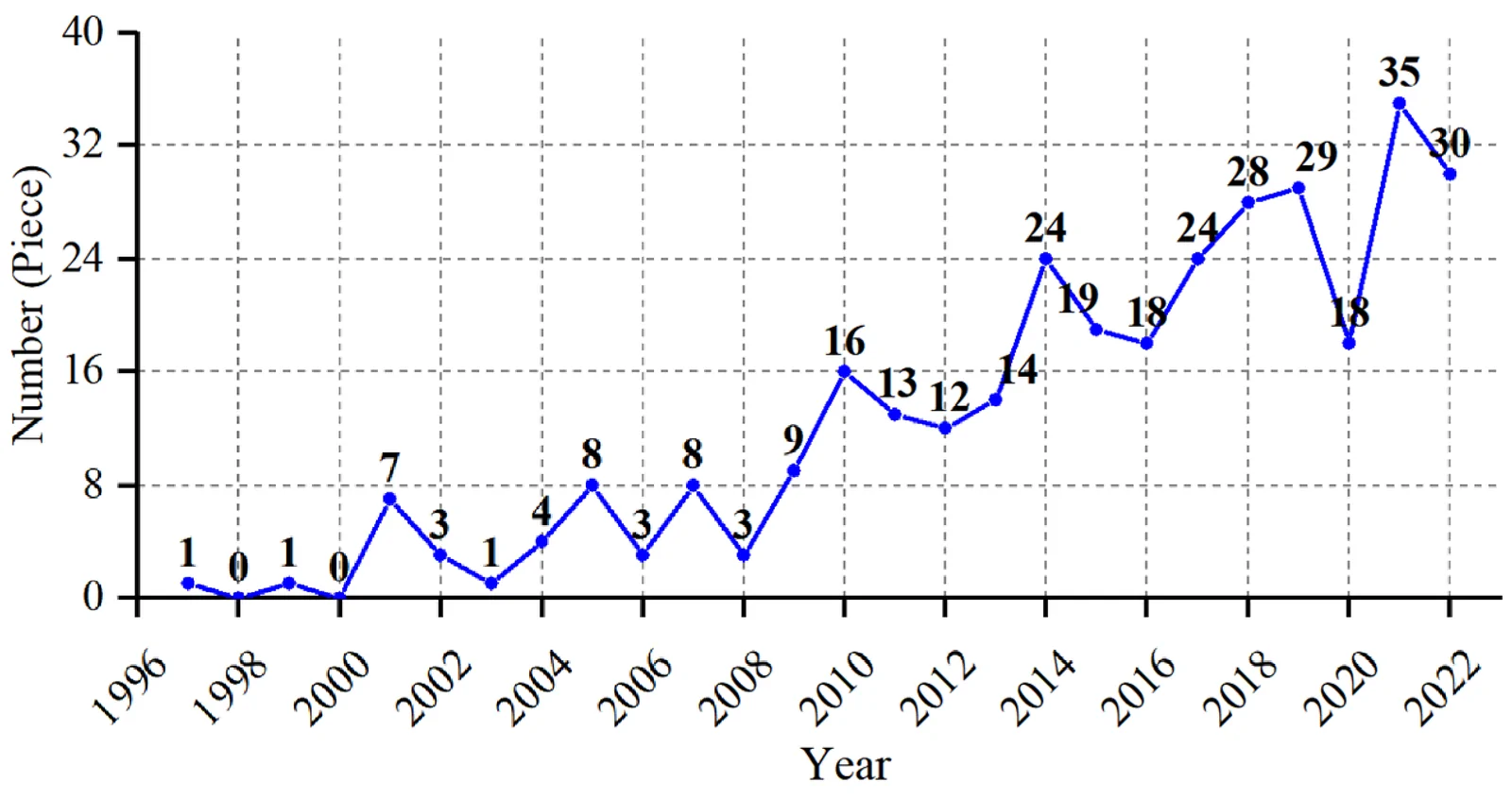
Publication trend in subjective value research.

Figure 2 illustrates the annual publication trend in subjective value research between 1997 and 2022. During the early stage of the field (1997–2009), the annual publication output remained relatively low, with fewer than ten publications per year. However, beginning around 2010, the field entered a period of sustained growth, culminating in its highest annual publication volume in 2021.

The rapid literature expansion reflects important theoretical and methodological advances in the study of judgment and decision-making under uncertainty. A key milestone was the introduction of cumulative prospect theory (Tversky & Kahneman, 1992), which provided a rigorous framework for quantifying subjective value under risk by incorporating probability weighting and loss aversion. Compared with earlier behavioral measures such as willingness to pay (WTP) and certainty equivalents (CEs), this framework enabled researchers to estimate latent subjective value more directly and systematically (Novemsky & Kahneman, 2005; Thaler, 1980).

Subsequent conceptual developments further accelerated research in this area. The value-based decision framework proposed by Rangel et al. (2008) and Glimcher’s (2011) two-stage model of decision-making established subjective value as the central cognitive representation linking information evaluation to behavioral choice. These influential models shifted research attention from observable decision outcomes to the valuation processes that precede choice, thereby stimulating extensive behavioral and neuroscientific investigations into how subjective value is constructed under conditions of risk, ambiguity, and uncertainty.

The 305 sample publications were distributed across 77 academic journals, reflecting the interdisciplinary nature of subjective value research. Table 3 presents the 10 journals publishing the largest number of articles and the 10 journals with the highest impact factors in the dataset. Collectively, the top 10 publishing journals account for 40.3% of all included articles, suggesting that research on subjective value has developed a well-defined core group of publication outlets.

These journals span multiple disciplines, including judgment and decision-making, behavioral science, psychology, management, neuroscience, and multidisciplinary science, highlighting the broad relevance of subjective value across behavioral and social sciences. Notably, the field is represented not only in specialized journals dedicated to decision-making research, but also in several high-impact, general interest journals. This publication pattern underscores the growing recognition of subjective value as a fundamental construct for understanding the cognitive and neural mechanisms underlying judgment and decision-making under conditions of risk, ambiguity, and uncertainty.

#### 3.2.2. Analysis of Core Authors and Core Author Groups

**Figure 3:**
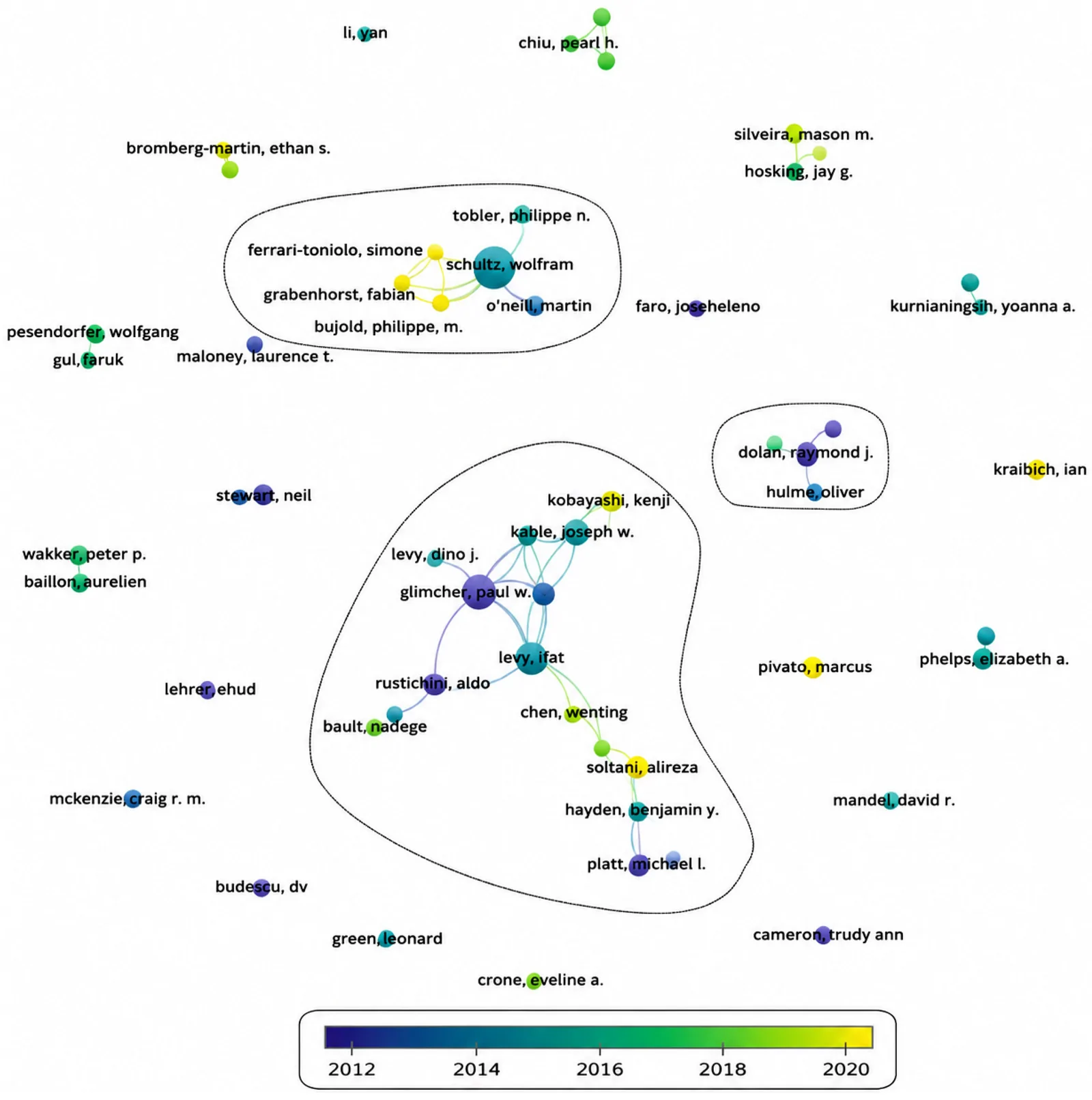
Core authors and core author groups in the field of subjective value research.

Analysis of the core author network provides insights into the principal contributors, collaborative structures, and the evolution of research on subjective value. To identify the field’s core research community, we used VOSviewer to construct a co-authorship network including 57 authors, each with at least two publications in the final sample (Parameter settings were as follows: Type of analysis = Co-authorship; Unit of analysis = Authors; Counting method = Full counting; Select “Ignore documents with a large number of authors”, and “Maximum number of authors per document” were retained at their default settings (25 authors); Minimum number of documents of an author = 2; Minimum number of citations of an author = 0; Number of authors to be selected = 57; Show all items). An overlay visualization was further applied to incorporate the temporal dimension of research activity. The resulting co-authorship network is presented in Figure 3.

In the network, node size is proportional to an author’s publication output, with larger nodes indicating a greater number of publications. Links between nodes represent co-authorship relationships, with connected authors having collaborated on one or more publications. Node color reflects the average publication year of each author’s work: lighter colors indicate more recent research activity, whereas darker colors represent earlier contributions. Three different black circles represent the three major research groups in the field. Together, these visual features reveal the field’s major research groups, patterns of scientific collaboration, and the temporal evolution of influential scholarship on subjective value.

**(1)**  **Core author analysis**

The core authors were calculated using the Price law, which is commonly used in bibliometrics. It is calculated using the formula $M=0.749\sqrt{N_{\max}}$, where *N*_max_ represents the number of publications by the author with the highest number of publications in the field. The number of publications by core authors should be greater than or equal to *M* (Borgohain et al., 2024). The scholar with the highest number of publications in this field published nine papers. After calculation, the core authors in this field should have at least 2.274 publications. In our dataset, there are 16 core authors with a total of 66 published papers, accounting for 21.63% of the total, which is lower than the Price law standard of 50%. However, if we round 2.274 down to 2 and count the number of authors with two or more publications, the number of publications by these authors accounts for 51.8% of the total. Combining the statistical data with Figure 3, it is clear that while the core group of authors in this field has already formed, the number of authors is small, while other author groups are numerous and highly promising. Information on the highly productive authors with four or more publications is summarized in Table 4.

**(2)**  **Core author groups**

Combining Figure 3 and Table 4 reveals a well-defined but relatively concentrated collaborative structure, with three major research clusters centered on W. Schultz (University of Cambridge), P. W. Glimcher (New York University), and R. Dolan (University College London). Importantly, these clusters are not simply groups of frequent collaborators; they represent three complementary intellectual trajectories through which subjective value research has developed from neural valuation mechanisms toward increasingly integrative accounts of judgment under uncertainty.

The Schultz cluster, including frequent collaborator P. N. Tobler, primarily represents the neural and computational foundations of valuation. This research stream examines how the brain encodes reward, probability, and subjective value and whether comparable valuation mechanisms operate across risky, certain, and intertemporal decisions. Its significance lies in establishing the biological plausibility of subjective value as an internal representation rather than merely a construct inferred from observed choice. By comparing valuation across decision contexts and competing theoretical models, this cluster also provides an important bridge between behavioral decision theory and neural measurement.

The Glimcher cluster, including I. E. Levy and J. W. Kable, most clearly represents the field’s movement toward an integrative computational framework. Its central contribution is the conceptualization of subjective value as a common neural currency that permits comparison across risky, ambiguous, intertemporal, informational, and consumer decisions. Research within this cluster also examines heterogeneity in risk and ambiguity preferences and their neural correlates. Closely related work by M. L. Platt extends this perspective to the evolutionary and adaptive foundations of risk preference. The prominence and breadth of this cluster therefore support one of our central conclusions: subjective value has increasingly evolved from a domain-specific measure of preference into a candidate cross-domain mechanism linking valuation to choice. At the same time, the diversity of decision contexts studied within this cluster raises an unresolved question about whether a common neural currency is genuinely domain-general or remains sensitive to task and context.

The Dolan cluster represents a complementary cognitive–affective perspective. In addition to examining the orbitofrontal cortex, striatum, and other neural systems involved in valuation, this research stream investigates how confidence, pessimism, emotion, and related psychological processes modify value-based judgment. Its significance is that it challenges an exclusively computational account of subjective value by demonstrating that valuation under uncertainty is dynamically shaped by cognitive and affective states.

Taken together, the three clusters reveal an important intellectual progression in the field: from identifying the neural mechanisms of value representation (Schultz), to constructing cross-domain computational accounts of subjective value (Glimcher), and to incorporating cognitive and affective influences on valuation (Dolan). This structure reinforces our theoretical conclusion that subjective value should not be understood solely as an economic utility parameter or an isolated neural signal, but as a multilevel construct connecting behavioral, computational, cognitive–affective, and neural processes. At the same time, the concentration of collaboration within three predominantly neuroscience-oriented clusters reveals a limitation of the field: interdisciplinary integration remains uneven. Greater collaboration among neuroscience, psychology, economics, and applied behavioral sciences is therefore needed to test whether current models of subjective value generalize across individuals, decision domains, and different forms of uncertainty.

#### 3.2.3. Highly Cited References

**Figure 4:**
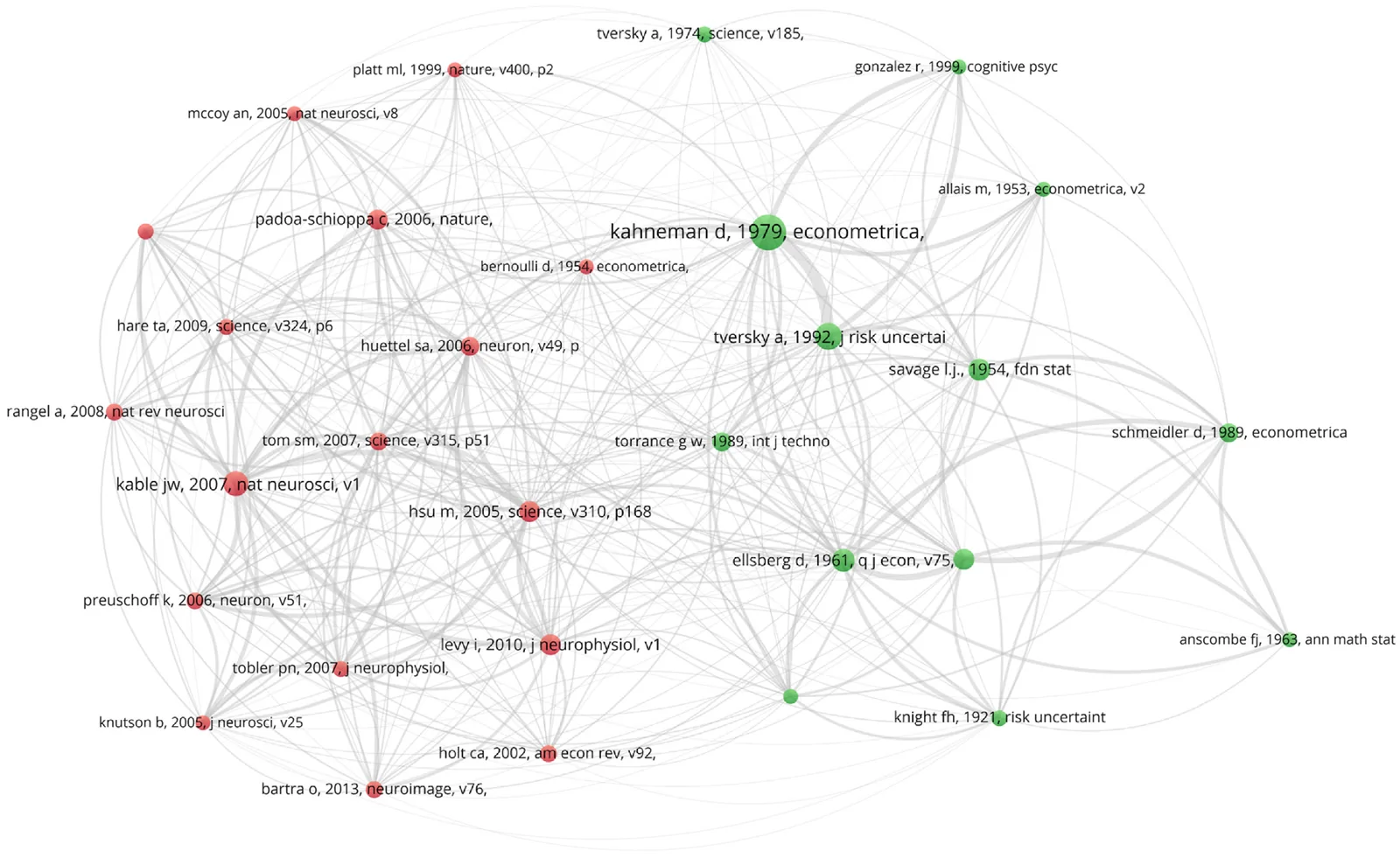
Co-citation map for subjective value studies.

Co-citation analysis identifies the intellectual foundations of a research field by revealing the publications that are most frequently cited together. To map the knowledge base of subjective value research, VOSviewer was used to analyze the co-citation network of references cited 14 or more times in the final sample (Parameter settings were as follows: Type of analysis = Co-citation; Unit of analysis = Cited references; Counting method = Full counting; Minimum number of citations of a cited reference = 14; Number of cited references to be selected = 30). The resulting network comprised 30 highly cited publications and is presented in Figure 4. In the figure, node size represents the number of co-citations, link thickness indicates the strength of co-citation relationships, and node color denotes clusters of closely related references. The 11 most highly cited publications are summarized in Table 5.

As shown in Figure 4, the co-citation network comprises two major knowledge clusters that reveal the interdisciplinary foundations of subjective value research. The green cluster represents economic and behavioral theories of decision-making under uncertainty, whereas the red cluster represents the neuroscientific foundations of subjective valuation. Their close connections indicate that the field has developed through an increasing integration of behavioral models explaining how subjective value should be represented with neuroscientific evidence identifying how valuation is implemented in the brain.

The green cluster is anchored by subjective expected utility theory (Savage, 1954), prospect theory (Kahneman & Tversky, 1979), cumulative prospect theory (Tversky & Kahneman, 1992), Maxmin Expected Utility Theory (Gilboa & Schmeidler, 1989), and Schmeidler’s (1989) work on subjective probability. Collectively, these theories established the behavioral foundations for modeling how individuals transform outcomes and probabilities into subjective valuations, while progressively distinguishing decisions under risk from those under ambiguity.

The red cluster provides the neural evidence that extended these theoretical constructs beyond observable choice. Hsu et al. (2005) demonstrated that ambiguity and expected reward engage partly distinct neural responses, while Padoa-Schioppa and Assad (2006) showed that orbitofrontal neurons encode abstract value rather than specific actions. Huettel et al. (2006) further identified partially distinct neural mechanisms underlying risk and ambiguity preferences. In contrast, Kable and Glimcher (2007) provided evidence for a common neural currency across decision domains, and Levy et al. (2010) showed that risk and ambiguity recruit shared valuation regions but that ambiguity additionally engages a broader neural network.

Overall, this co-citation structure reveals a central theoretical tension rather than simple convergence. Behavioral theories increasingly distinguish risk from ambiguity, and some neural evidence supports this distinction (Hsu et al., 2005; Huettel et al., 2006), whereas common currency research emphasizes shared valuation mechanisms across forms of choice (Kable & Glimcher, 2007; Levy et al., 2010). Thus, Figure 4 reinforces our conclusion that subjective value provides an important bridge between behavioral and neural accounts of decision-making, while highlighting an unresolved question: to what extent does the brain rely on a domain-general valuation mechanism versus uncertainty-specific processes? Resolving this tension represents a central priority for future neuroeconomic research.

Importantly, these foundational studies continue to shape contemporary research on judgment and decision-making under uncertainty. Hsu et al. (2005), Padoa-Schioppa and Assad (2006), and Huettel et al. (2006) established influential experimental paradigms for investigating uncertainty and valuation, whereas Levy et al. (2010) provided the first systematic examination of subjective value under both risk and ambiguity, becoming a cornerstone of neuroeconomic research. Meanwhile, Kable and Glimcher (2007) extended the concept of subjective value beyond uncertainty and reinforced the view that subjective value functions as a general-domain neural currency that supports diverse forms of judgment and choice.

### 3.3. Research Themes in Subjective Value and Decision-Making Under Uncertainty

#### 3.3.1. Identifying Core Research Themes

**Figure 5:**
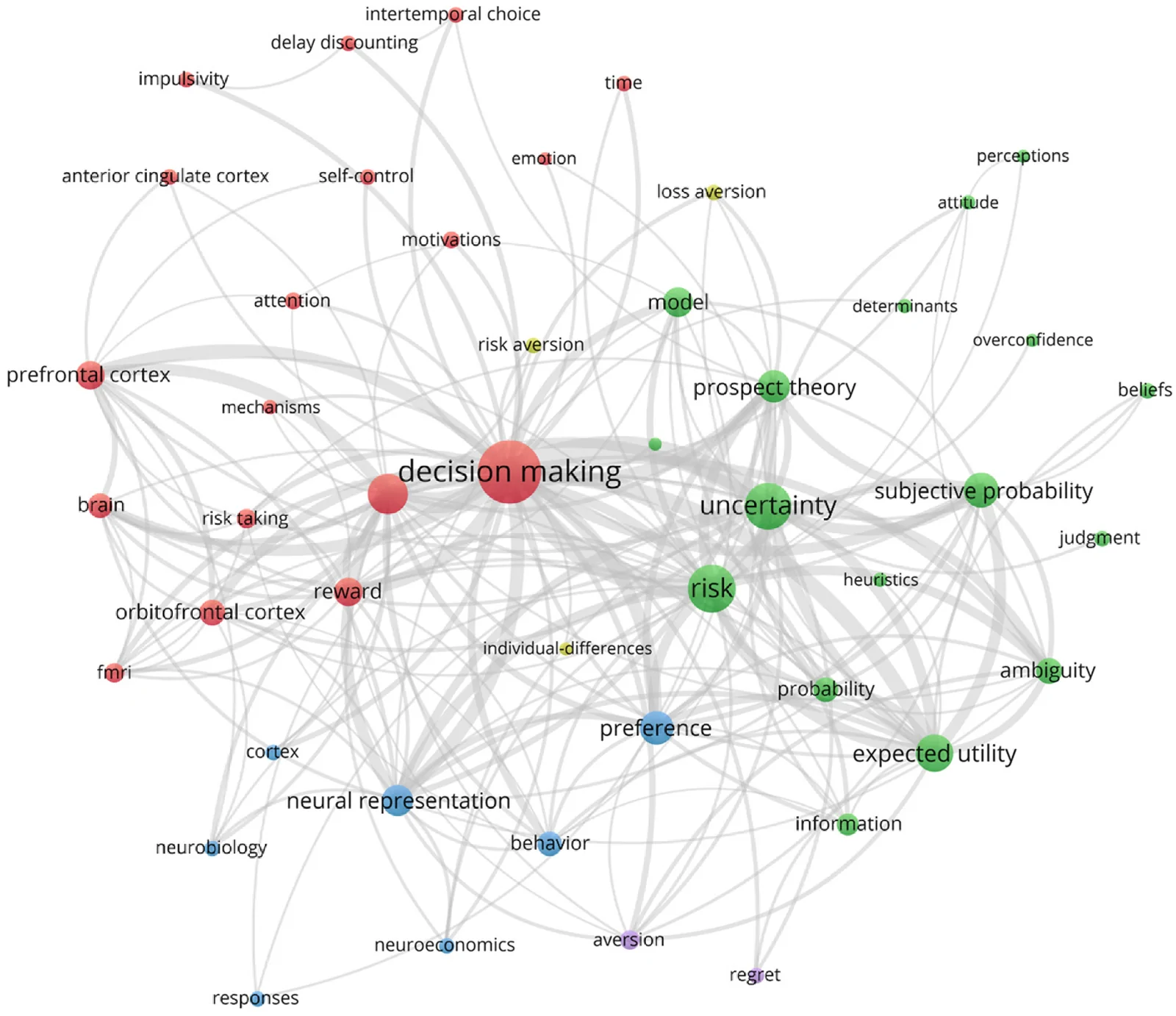
Keyword co-occurrence mapping of subjective value studies.

Keyword co-occurrence analysis was conducted to identify major research themes and the conceptual structure of subjective value research in judgment and decision-making under uncertainty. Prior to analysis, keywords with equivalent meanings were standardized and merged (“decision making”, “decision-making”, “decision” and “choice” to “decision making”; “brain” and “human brain” to “brain”; “subjective probability” and “subjective-probability” to “subjective probability”; “preference” and “preferences” to “preference”; “prospect theory” and “prospect-theory” to “prospect theory”; “neural representation” and “representation” to “neural representation”; “prefrontal cortex” and “ventromedial prefrontal cortex” to “prefrontal cortex”; “value” and “values” to “value”) to improve consistency. A total of 47 keywords with a minimum occurrence frequency of seven were retained for analysis using VOSviewer. The keyword co-occurrence network is presented in Figure 5 (Parameter settings were set as follows: Type of analysis = Co-occurrence; Unit of analysis = All keywords; Counting methods = Full counting; Minimum number of occurrences of a keyword = 7; Number of keywords to be selected = 57; Select “Network visualization” option), while the most frequently occurring keywords are reported in Table 6.

In the co-occurrence network, node size represents the frequency at which a keyword appears in the literature, with larger nodes indicating greater research attention. Links between nodes represent the strength of keyword co-occurrence, where thicker links indicate stronger conceptual associations between research topics. Node colors denote clusters of closely related keywords that represent distinct research themes. After removing small clusters containing three or fewer keywords, three major thematic clusters emerged.

These clusters provide a systematic overview of the field’s intellectual structure and reveal the primary directions of research on subjective value. More importantly, the network illustrates how studies have evolved from examining behavioral responses to uncertainty toward investigating the cognitive and neural mechanisms through which subjective value is constructed under conditions of risk, ambiguity, and uncertainty.

The three clusters represent the major research themes in subjective value research while also revealing several unresolved theoretical and methodological tensions. Theme 1 (green cluster) focuses on the behavioral foundations of decision-making under uncertainty, including competing models of valuation, probability judgment, and behavioral responses to risk and ambiguity. Despite substantial theoretical development, disagreement remains regarding how subjective value should be modeled across different forms of uncertainty and whether risk-based models can adequately capture valuation under ambiguity. Theme 2 (red cluster) centers on the neural representation of subjective value, examining the brain mechanisms underlying valuation during risky and ambiguous decisions. Although considerable evidence supports a common neural valuation system, findings concerning the functions and specificity of individual brain regions remain heterogeneous across tasks and decision contexts. Theme 3 (blue cluster), led primarily by Glimcher and colleagues, seeks to reconcile these perspectives by mapping behavioral and computational constructs onto their neural representations. However, whether subjective value can function as a genuinely domain-general “common currency” and whether behavioral and neural measures capture the same underlying construct remain open questions. Thus, the three themes reflect not only complementary research streams but also continuing debates over the conceptualization, measurement, and neural implementation of subjective value under uncertainty.

##### Theme 1: Behavioral Foundations of Judgment and Decision-Making Under Uncertainty

Behavioral research provides a theoretical foundation for explaining how subjective value is constructed during judgment and decision-making under uncertainty. As illustrated in Figure 5 and Table 6 and Table 7, four interrelated research streams have emerged. Importantly, however, these streams also reveal continuing debates over how subjective value should be modeled, whether risk and ambiguity involve equivalent valuation processes, and how contextual information enters value construction.

First, the development and comparison of behavioral decision models remain central to the field. Prospect theory continues to dominate contemporary research, followed by expected utility theory, reflecting their enduring influence on subjective value estimation. Yet neither framework fully resolves how people make decisions when information is incomplete or cognitive resources are constrained. Heuristic approaches challenge the assumption that individuals routinely compute and compare integrated expected values. For example, Zhang et al. (2014) proposed the Equate-to-Differentiate Theory, suggesting that decision-makers may simplify comparisons rather than perform computationally demanding expectation calculations under time or cognitive constraints. The coexistence of utility-based and heuristic accounts raises an important theoretical question: whether subjective value is always computed as an integrated representation before choice or whether some decisions emerge from simpler comparison rules without full valuation. Thus, bounded rationality is not merely a complement to utility-based theories but potentially challenges their assumptions about the processes generating observed choices.

Second, substantial uncertainty also remains regarding how probabilities are psychologically represented. Although power function representations of outcome value are relatively established, no comparable consensus exists regarding the functional form of probability weighting. Li et al. (2018), for example, found that a two-parameter model capturing both probability attitude and sensitivity outperformed one-parameter alternatives and that probability sensitivity was particularly important under ambiguity. The Goldstein–Einhorn model also provided a better fit than the Prelec two-parameter and Neo-Additive models, especially for ambiguous decisions. Such model-dependent results raise an important methodological concern: estimates of subjective value may depend partly on the functional form imposed by researchers rather than solely on the underlying valuation process. Consequently, apparent individual differences in subjective value may reflect not only genuine heterogeneity in uncertainty preferences but also differences in model specification. Comparative model testing across tasks, populations, and uncertainty conditions is therefore necessary before any particular probability-weighting function can be treated as generalizable.

Third, an important theory–measurement tension concerns the relationship between risk and ambiguity. Research on risky decisions substantially exceeds research on ambiguous decisions, and ambiguity studies frequently adapt valuation models originally developed for known probabilities (Edelson et al., 2018; FeldmanHall et al., 2016; Levy et al., 2010; Williams et al., 2021). Although this approach facilitates comparison across uncertainty conditions, it implicitly assumes substantial continuity between the valuation processes underlying risk and ambiguity. This assumption remains debatable because ambiguous decisions require individuals to evaluate incomplete probability information rather than simply transform known probabilities. Contextual evidence further complicates the picture. Weber et al. (2002) demonstrated that risk-taking varies considerably across financial, health/safety, recreational, ethical, and social domains, while Li et al. (2018) found lower ambiguity avoidance and greater rationality in naturalistic than laboratory contexts. These findings challenge the treatment of uncertainty attitudes as stable, domain-general preferences and suggest that subjective value is partly constructed from the interaction between individual preferences and decision context. Developing ambiguity-specific models and testing their robustness across ecologically meaningful contexts therefore remain important priorities.

Fourth, the literature increasingly challenges the assumption that uncertainty is determined simply by the amount of missing information. Ellsberg (1961) associated ambiguity aversion with incomplete probability information, while Kahneman and Tversky (1979) linked the certainty effect to differences in probabilistic information. More recent evidence suggests a more complex process in which the psychological meaning of an information gap matters alongside its magnitude. Golman et al. (2021) showed that uncertainty can be either avoided or sought depending on whether information gaps are experienced as aversive or rewarding. Moreover, Pushkarskaya et al. (2015) found that individuals may respond more strongly to conflict within known probability information than to ambiguity itself, while Peysakhovich and Karmarkar (2016) demonstrated asymmetric effects of favorable and unfavorable information on subjective value. Charpentier et al. (2018) further showed that favorable information can possess intrinsic utility and motivate information seeking. Together, these findings question accounts that reduce ambiguity to missing probability information alone. Instead, subjective value appears to depend jointly on information availability, valence, conflict, and the affective significance of uncertainty.

Overall, the behavioral literature has progressed from treating subjective value as a relatively stable transformation of outcomes and probabilities toward viewing valuation as a context-sensitive psychological construction. Nevertheless, important controversies remain unresolved: whether choices always require integrated subjective value computation, which probability-weighting models best capture valuation, whether risk-derived models adequately represent ambiguity, and how informational and contextual factors reshape uncertainty preferences. Resolving these issues will be essential for developing behavioral models that are both theoretically precise and sufficiently flexible to explain judgment and decision-making across diverse forms of uncertainty.

##### Theme 2: Neural Representation of Subjective Value

A second major theme concerns the neural mechanisms through which subjective value is computed and represented during judgment under uncertainty. Functional magnetic resonance imaging (fMRI) remains the dominant method, complemented by event-related potentials (ERPs) (Wang et al., 2019) and causal approaches such as electrical stimulation (Ballesta et al., 2020, 2022). As shown in Figure 5 and Table 6 and Table 7, research has concentrated particularly on the ventromedial prefrontal cortex (vmPFC), orbitofrontal cortex (OFC), and anterior cingulate cortex (ACC). However, their precise and potentially overlapping functions remain debated.

The vmPFC is most consistently associated with subjective valuation. Tom et al. (2007) found that subjective value under risk is represented jointly in the vmPFC and ventral striatum, while subsequent studies identified the vmPFC as a domain-general valuation region across different goods and choice contexts (Abitbol et al., 2015; Chib et al., 2009; Clithero & Rangel, 2014). These findings support the common currency hypothesis, whereby heterogeneous options are converted into a comparable neural value signal. However, evidence of context-dependent valuation and distributed neural involvement cautions against interpreting vmPFC activation as a unique or context-invariant marker of subjective value.

The ACC and OFC appear to perform complementary functions in uncertain valuation. The ACC is particularly implicated in conflict monitoring and adaptation: its activity increases when competing options have similar values or valuation systems generate conflicting signals (Yeung et al., 2004), and it responds to environmental volatility that requires updating existing value estimates (Rushworth & Behrens, 2008). The OFC, in contrast, has been associated with uncertainty attitudes and value comparison. Orbitofrontal impairment can substantially reduce both risk and ambiguity aversion (Hsu et al., 2005), while OFC activity tracks economic valuation (Plassmann et al., 2007). Electrical stimulation studies in macaques provide stronger causal evidence linking OFC activity to value comparison and choice (Ballesta et al., 2020, 2022). Nevertheless, differences between human neuroimaging and animal intervention studies make direct functional equivalence difficult to establish.

Importantly, subjective value is not generated by a fixed valuation network alone but is dynamically shaped by cognitive and affective processes (Zhu & Wang, 2015). Self-control can modify vmPFC value signals through dorsolateral prefrontal activity (Hare et al., 2009), attention alters value representations in the vmPFC and ventral striatum (Lim et al., 2011), and both negative and positive emotions influence valuation and risk preference (Heilman et al., 2010; Winecoff et al., 2013). These findings challenge a purely localized account of subjective value and instead support a distributed, context-sensitive valuation process integrating value, cognitive control, attention, and affect.

Overall, neuroscience provides substantial evidence for identifiable neural systems underlying subjective valuation, but important methodological and theoretical uncertainties remain. The heavy reliance on correlational fMRI evidence limits causal inference, regional activation does not necessarily imply functional specificity, and findings may vary across tasks, species, and forms of uncertainty. Future research should therefore combine neuroimaging with temporally precise and causal methods and directly compare risk and ambiguity to determine which neural mechanisms are common across uncertainty and which are context-specific.

##### Theme 3: Integrating Behavioral and Neuroscientific Perspectives

The third theme seeks to integrate behavioral, computational, and neuroscientific evidence into a unified framework for judgment and decision-making under uncertainty. Glimcher (2011) proposed systematic mappings between economic constructs and neural processes, most notably between utility in economic theory and subjective value represented in the ventral striatum and medial prefrontal cortex. This framework shifts the study of choice from inferring preferences solely from observed behavior toward identifying the internal computations through which value is represented and translated into action.

This integrative approach is reinforced by Fehr and Rangel (2011), who advocated reciprocal validation between subjective value estimated from behavioral models and value signals observed at the neural level. Such convergence offers a powerful means of testing whether theoretical constructs correspond to underlying cognitive mechanisms. However, this correspondence should not be assumed. Behavioral estimates of subjective value are model-dependent, whereas neural measures are largely indirect and correlational. Similar behavioral choices may therefore arise from different valuation processes, while similar neural activity may support different computations across decision contexts. The distinction between revealed preference and subjective value is particularly important: whereas traditional economic approaches infer preferences from choices, neuroeconomic approaches seek to identify the latent valuation processes generating those choices (Glimcher, 2011; Jia, 2016).

Emerging evidence further suggests that individual differences in valuation may have structural as well as functional neural foundations. Gilaie-Dotan et al. (2014), for example, associated greater gray matter volume in the right posterior parietal cortex with less compressed neural value representations and greater risk tolerance. Such findings broaden cross-domain models by linking biological variation to behavioral heterogeneity.

**Figure 6:**
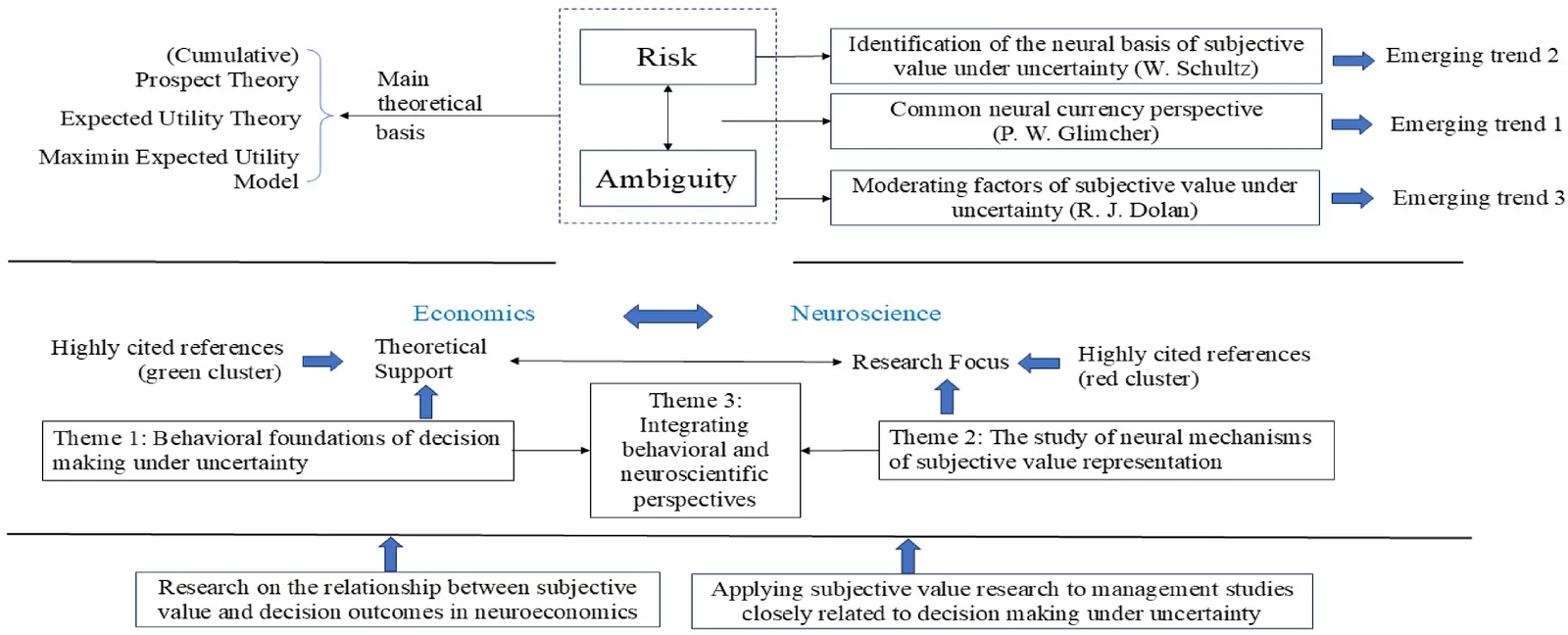
An integrated overview of subjective value studies. **Note**. “Highly cited references (green cluster)” and “Highly cited references (red cluster)” represent the clustering results presented in Section 3.2.3 (Figure 4). Specifically, the “green cluster” represents the economic foundations of the field, corresponding to the green cluster shown in Figure 4. The “red cluster” represents the neuroscientific foundations of the field, corresponding to the red cluster shown in Figure 4.

Overall, cross-domain integration offers considerable potential for a mechanistic account of decision-making under uncertainty, but a fully unified model remains premature. Future research should establish more precise mappings among economic constructs, behavioral parameters, cognitive processes, and neural mechanisms, while testing whether these mappings remain stable across risk, ambiguity, individuals, and decision contexts. Such work is essential for determining whether subjective value constitutes a genuinely domain-general mechanism or a context-sensitive representation constructed during judgment. Building on these established foundations, subjective value can be investigated from a broader perspective. For example, examining its relationship with decision outcomes may provide deeper insights into the neural basis of the gap between valuation and behavior. Such insights may also extend beyond neuroeconomics to management domains closely related to decision-making under uncertainty, such as leadership and strategy (please see Figure 6 for an integrated overview of subjective value studies).

#### 3.3.2. Emerging Trends and Future Research Directions

**Figure 7:**
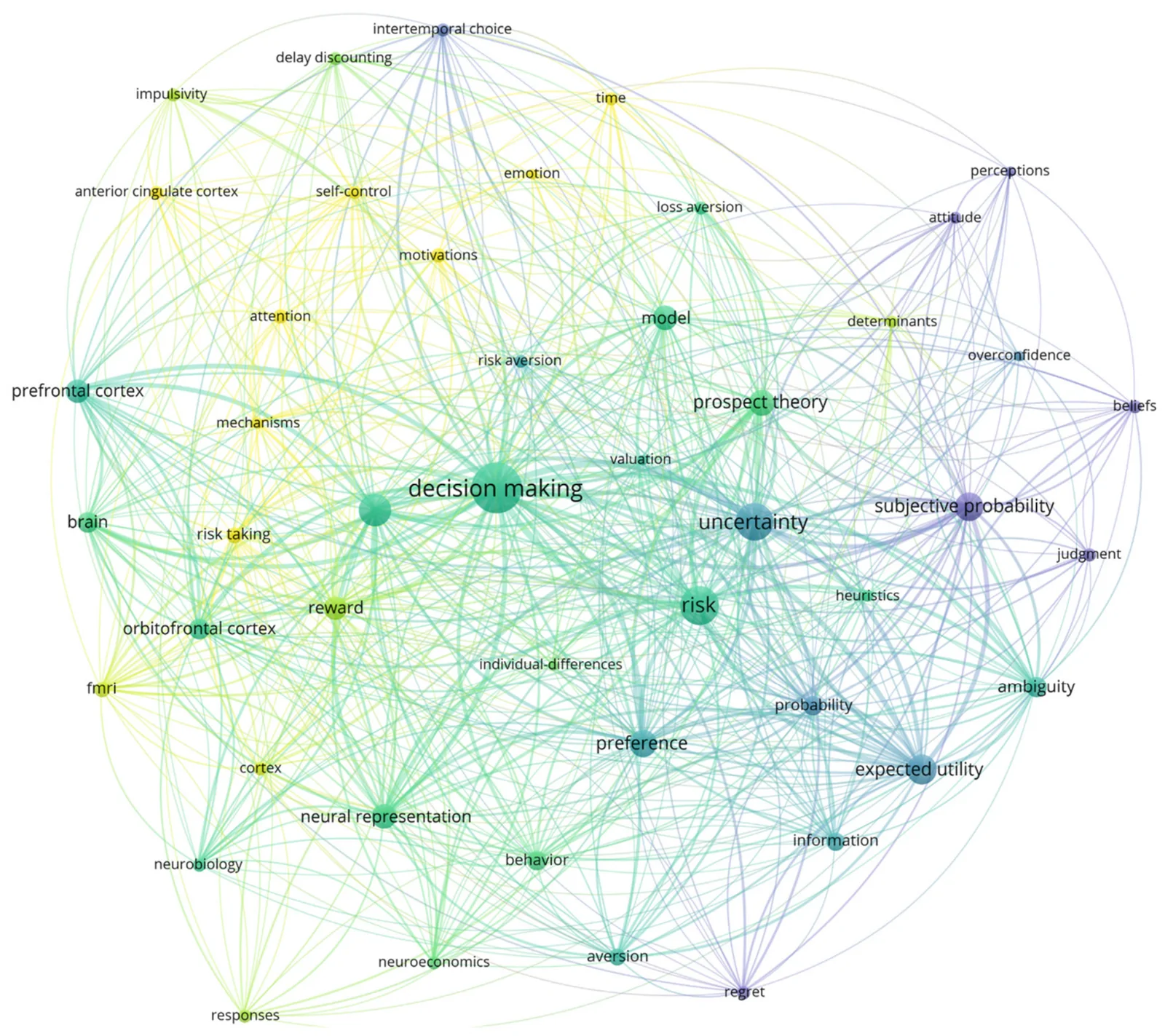
Keyword co-occurrence superposition with time for subjective value research.

Overlay visualization of the keyword co-occurrence network provides insights into the temporal evolution of research topics. For this analysis, we applied the same keyword standardization procedure as described in Section 3.3.1. Parameter settings were as follows: Type of analysis = Co-occurrence; Unit of analysis = All keywords; Counting methods = Full counting; Minimum number of occurrences of a keyword = 7; Number of keywords to be selected = 57; Select “Overlay visualization” option. In Figure 7, node color represents the average publication year of the corresponding keyword, with lighter colors indicating more recent research attention. Because recently emerging topics may have lower overall frequencies, both keyword occurrence (Table 6) and temporal information (Figure 7) were considered when identifying frontier research trends.

Three major trends emerged. First, among the early theoretical keywords, risk, ambiguity, prospect theory, and model remain highly active, indicating that theoretical research on judgment and decision-making under uncertainty continues to evolve. By contrast, the keyword uncertainty has appeared less frequently in recent years, reflecting an increasing conceptual distinction between risk and ambiguity following Knight’s (1921) framework. Earlier studies often used uncertainty as a generic term for risky decisions, whereas recent research has increasingly recognized ambiguity as a distinct form of uncertainty requiring separate theoretical and empirical investigation. Meanwhile, the declining prominence of subjective probability suggests that, although its functional form remains debated, research has gradually converged on its conceptualization and measurement (Edelson et al., 2018; Williams et al., 2021).

Second, with the rapid development of neuroeconomics, keywords such as neural representation, prefrontal cortex, and orbitofrontal cortex have become increasingly prominent. This trend reflects growing interest in uncovering the neural mechanisms through which subjective value is computed and integrated during judgment under uncertainty (Dennison et al., 2022; Hsu et al., 2005; Levy et al., 2010).

Third, recent studies have increasingly emphasized individual cognitive and affective processes. Risk-taking has become one of the fastest-growing topics, while emotion, attention, and self-control have also emerged as important themes. These developments are consistent with the broader shift in behavioral decision-making research toward understanding how individual differences and psychological processes shape judgments under conditions of risk and ambiguity (Chung et al., 2015; Dennison et al., 2022; Phelps et al., 2014).

Therefore, the following four themes are promising directions for future research.

First, future studies should further develop theories of subjective value under ambiguity. Although substantial progress has been made in modeling decisions under risk, subjective value under ambiguity remains comparatively underdeveloped. Previous studies have focused primarily on the degree of ambiguity, whereas the role of known information—including its positive and negative valence—has received much less attention. Future studies should investigate how individuals integrate known and unknown information during valuation, examine how subjective value evolves as information gaps are reduced, and develop experimental paradigms that more closely reflect real-world ambiguous decisions. In addition, many current studies have estimated subjective value under ambiguity using computational models originally developed for risky decision-making (Edelson et al., 2018; FeldmanHall et al., 2016; Levy et al., 2010; Williams et al., 2021). Given the distinct psychological processes underlying risk and ambiguity, future research should develop valuation models specifically tailored to ambiguous decisions. Initial efforts in this direction have already been reported by Li et al. (2018), who compared alternative subjective value models across risky and ambiguous decision contexts.

Second, future research should clarify the relationship between subjective value and decision outcomes. Contemporary decision process models distinguish value computation from behavioral choice (Glimcher, 2011; Rangel et al., 2008), yet empirical studies often infer subjective value directly from observed decisions (Fehr & Rangel, 2011). Laboratory decisions typically occur under relatively unconstrained conditions, whereas real-world choices are shaped by external constraints such as time pressure, financial limitations, institutional rules, and social norms (McFadden, 2001). In many cultural contexts, including China, additional social considerations such as face concern and compromise may further influence the translation of subjective value into observable decisions (Ma, 2010; Ma et al., 2015). Examining these contextual constraints as boundary conditions would improve our understanding of how internal valuation processes are converted into actual judgments and choices, thereby strengthening the ecological validity of subjective value research.

Third, greater integration of subjective value research within applied decision-making contexts is needed. The growing prominence of risk-taking suggests increasing opportunities to extend neuroeconomic theories to organizational and managerial decision-making. Building on the behavioral strategy framework proposed by Powell et al. (2011), future studies could integrate theories of subjective value with strategic decision-making under uncertainty. Recent applications of subjective value paradigms to leadership (Edelson et al., 2018) and prospective decision-making (Soutschek et al., 2021) demonstrate the feasibility of combining neuroeconomic methods with management research. Extending these approaches to domains such as organizational behavior, entrepreneurship, and strategic management would enhance the practical relevance of subjective value research while enriching theories of judgment under uncertainty.

Finally, future research should continue refining cross-domain models in neuroeconomics. Integrating behavioral, computational, and neuroscientific evidence has substantially advanced our understanding of judgment under uncertainty. Nevertheless, inconsistencies remain across existing cross-domain frameworks, as discussed in Section 2.2. In particular, several important concepts from behavioral economics, such as expected noise, have not yet been clearly mapped onto their neural representations. Establishing stronger conceptual correspondence between economic constructs, cognitive processes, and neural mechanisms will be essential for developing more comprehensive interdisciplinary models of judgment and decision-making under uncertainty.

## 4. Conclusions, Limitations, and Implications

Subjective value has emerged as a core construct for understanding how individuals form judgments and make decisions under conditions of risk, ambiguity, and uncertainty. By integrating evidence from behavioral decision theory, economics, and neuroscience, this study demonstrates that research on subjective value has developed a robust interdisciplinary foundation. Prospect theory and cumulative prospect theory provide the principal theoretical basis for modeling subjective value, while advances in neuroeconomics have revealed the cognitive and neural mechanisms through which subjective value is computed and translated into behavioral choice. The concentration of publications in leading journals in decision science, behavioral science, psychology, management, neuroscience, and multidisciplinary research further highlights the growing importance of this field.

Several limitations should be acknowledged for this study. First, the bibliometric analysis relied exclusively on the Web of Science Core Collection and included only English-language journal articles and reviews. This sampling strategy may introduce publication and database selection bias by underrepresenting relevant studies published in other databases, languages, books, conference proceedings, or unpublished sources. Moreover, bibliometric indicators such as citation frequency and keyword co-occurrence may favor older, highly cited, or more extensively studied topics, while emerging or less mainstream perspectives may receive comparatively limited visibility. Although manual screening and qualitative synthesis were used to improve relevance and interpretation, researcher judgment in study selection and keyword consolidation may also introduce subjectivity. In addition, the literature included in this study was collected up to the end of 2022. In recent years, artificial intelligence tools have increasingly influenced neuroeconomic research by facilitating behavioral data fitting and neural data analysis. These technological advances may lower methodological barriers and attract more researchers with backgrounds in economics and related fields to contribute to neuroeconomics. Consequently, emerging studies published after 2022 may introduce new research themes and methodological approaches that are not fully captured in the present review, potentially limiting the accuracy of identifying recent research trends. Future reviews could address these limitations by integrating multiple databases, broader publication types and languages, and complementary systematic review or meta-analytic methods.

Our findings have implications for the future development and application of subjective value research. First, for neuroeconomics, the concentration of the field within neuroscience-oriented author clusters indicates a need for deeper integration with economics, psychology, and behavioral decision science. The theoretical synthesis in Section 2 can help neuroscientists connect neural evidence more explicitly to economic theories of valuation, while the bibliometric mapping in Section 3 enables behavioral and economic researchers to identify relevant neuroscientific mechanisms, methods, and emerging research opportunities. Second, for behavioral decision-making research, our findings highlight the need to move beyond observable choices toward understanding how subjective value is shaped by ambiguity, information valence, emotion, attention, and contextual constraints. Third, these insights have potential public policy implications because policies communicating uncertain information—for example, in health, finance, and public risk—may influence behavior not only through objective probabilities but also through how individuals subjectively value and interpret uncertainty. Finally, for organizational decision-making, subjective value provides a useful framework for explaining heterogeneous responses to uncertain strategic alternatives, particularly when managers face incomplete information, competing outcomes, and changing environments. Greater integration of behavioral, economic, and neuroscientific perspectives can therefore advance both the explanatory power and the real-world relevance of research on judgment and decision-making under uncertainty.

## Figures and Tables

**Table 1:** Conceptual expressions of the subjective value of decision-making under uncertainty.

Subjective Value Dimension		Concept Essence	Conceptual Expressions Commonly Used in the Literature
Outcome Level	Potential gains	Potential gains’ subjective value	Value (in gain domain)
	Potential losses	Potential losses’ subjective value	Value (in loss domain)
Option-Level		Option’s expected subjective value	Subjective value
Choice-Level	Mixed binary	Expected subjective value difference	(Gamble’s/lottery’s) subjective value
	Loss binary	Expected subjective value difference	(Gamble’s/lottery’s) subjective value difference
	Gain binary		

**Table 2:** Evolution of subjective value and theoretical foundations of decision-making under uncertainty.

Theory or Important Work	Concept Closely Related to Subjective Value	Subjective and Objective Properties of Basic Logical Units		Phenomena that Cannot Be Explained or Violations of the Basic Principles of Economics	Research Basis	Progress Compared to Research Basis
		Potential Gains/Losses	Probability			
Expected value theory (Pascal, 1670, 1966)	Expected value	Objective	Objective	St. Petersburg paradox	——	The first theory to propose two basic logical units for the valuation of uncertain decision-making options.
Bernoulli principle (Bernoulli, 1738, 1954)	Utility	Subjective	Objective	——	Expected value theory	Proposes subjective properties for potential gain/loss value judgments and explains the St. Petersburg paradox.
Expected utility theory (Von Neumann & Morgenstern, 1944)	Expected utility	Subjective	Objective	Allais paradox, Ellsberg’s paradox, etc.	Bernoulli principle	Development from cardinal utility theory to ordinal utility theory and construction of an axiomatic system of ordinal utility theory.
Incorporating subjective probabilities into normative decision systems Ramsey (1931)	——	Subjective	Subjective	——	——	Defines probabilistic judgments with subjective properties; suggests that there is no need to distinguish between risky and ambiguous decision-making.
Finetti’s development of subjective probabilities (Finetti, 1931)	——	Subjective	Subjective	——	Axiomatic framework of subjective expected utility	Support through mathematical arguments.
Subjective expected utility theory (Savage, 1954)	Subjective expected utility	Subjective	Subjective	Ellsberg’s paradox	Expected utility theory; subjective probability theory	Blending of the two views.
Prospect theory (Kahneman & Tversky, 1979)	Value	Subjective	Subjective	First-order stochastic dominance	Expected utility theory	Considers subjective value judgment to be related to reference points; explains the Allais paradox, Ellsberg’s paradox, etc.
A series of rank dependency theories (Quiggin, 1982)	——	No Emphasis	Subjective	Insufficient consideration of potential outcomes, resulting in insufficient robustness	Expected utility theory; subjective expected utility theory	Separation of ambiguity and risk modeling studies; distinguishes between decision weights and probability weights.
Cumulative prospect theory (Tversky & Kahneman, 1992)	Subjective value	Subjective	Subjective	——	Prospect theory; rank-dependent expected utility theory	Integration of two theories; proposes a specific functional form for the value function and the weight function.
Theoretical neuroeconomic framework for uncertainty decision-making (Glimcher, 2011)	Subjective value	No Emphasis	No Emphasis	Conflicting perceptions of rational/irrational; based on cardinal utility theory; at odds with mainstream economic views	Expected utility theory; related neurobiological theories	Introduces uncertainty decision-making to the field of neuroscience.

**Table 3:** Top 10 journals with the highest number of subjective value publications and the highest impact factor.

Rank	Top 10 Journals Based on Number of Articles Published		Top 10 Journals Based on Impact Factor	
	Journal	N	Journal	N
1	*Journal of Neuroscience*	21	*Nature Neuroscience*	4
2	*Frontiers in Psychology*	21	*Trends in Cognitive Sciences*	2
3	*Economic Theory*	12	*Academy of Management Review*	1
4	*PNAS*	12	*Academy of Management Journal*	2
5	*Frontiers in Neuroscience*	11	*Nature Communications*	3
6	*Journal of Economic Behavioral Decision Making*	11	*Nature Human Behavior*	3
7	*Journal of Risk and Uncertainty*	10	*PNAS*	12
8	*Journal of Behavioral Decision Making*	9	*American Economic Review*	1
9	*Management Decision*	8	*Management* *Science*	7
10	*Theory and Decision*	8	*Organizational Behavioral and Human Decision Processes*	5

**Note**. The commonly used abbreviation “*PNAS*” is used in the table to refer to the journal *Proceedings of the National Academy of Sciences* from the United States of America.

**Table 4:** List of highly productive researchers in the field of subjective value research.

Rank	Author	Number of Articles Published	Affiliated Institution
1	Schultz, Wolfram	9	University of Cambridge
2	Glimcher, Paul W.	7	New York University
3	Levy, Ifat	7	University of Haifa
4	Dolan, Raymond J.	4	University College London
5	Platt, Michael L.	4	University of Pennsylvania
6	Tobler, Philippe N.	4	University of Zurich
7	Kable, Joseph W.	4	University of Pennsylvania

**Table 5:** List of highly cited studies on subjective value research.

Frequency	Year	First Author	Title of the Article	Journal
70	1979	KAHNEMAN D	Prospect Theory: An Analysis of Decision under Risk	*Econometrica*
42	1992	TVERSKY A	Advances in Prospect Theory: Cumulative Representation of Uncertainty	*Journal of Risk and Uncertainty*
36	2007	KABLE J W	The Neural Correlates of Subjective Value During Intertemporal Choice	*Nature Neuroscience*
31	1961	ELLSBERG D	Risk, Ambiguity, and the Savage Axioms	*The Quarterly Journal of Economics*
28	1954	SAVAGE L J	The Foundations of Statistics	*John Wiley*
26	2005	HSU M	Neural Systems Responding to Degrees of Uncertainty in Human Decision-Making	*Science*
26	2010	LEVY I	Neural Representation of Subjective Value under Risk and Ambiguity	*Journal of Neurophysiology*
25	1989	GILBOA I	Maxmin Expected Utility with Non-Unique Prior	*Journal of Mathematical Economics*
23	2006	PADOA-SCHIOPPA C	Neurons in the Orbitofrontal Cortex Encode Economic Value	*Nature*
21	2006	HUETTEL S A	Neural Signatures of Economic Preferences for Risk and Ambiguity	*Neuron*
21	1989	TORRANCE G W	Utilities and Quality-Adjusted Life Years	*International Journal of Technology Assessment in Health Care*
21	1989	SCHMEIDLER D	Subjective Probability and Expected Utility without Additivity	*Journal of the Econometric Society*

**Table 6:** List of high-frequency keywords in subjective value studies.

Keyword	Frequency	Keyword	Frequency	Keyword	Frequency
Decision making	132	Prospect theory	37	Brain	23
Risk	79	Neural representation	34	Probability	22
Uncertainty	74	Model	33	Behavior	22
Subjective value	57	Reward	29	Information	19
Expected utility	49	Prefrontal cortex	29	Aversion	15
Subjective probability	42	Ambiguity	25	fMRI	14
Preference	39	Orbitofrontal cortex	24	Risk-taking	14

**Table 7:** Themes in subjective value studies and the representative keywords for each theme.

	Research Themes	Representative Keywords
1	Theoretical studies on decision-making behaviors under uncertainty and subjective value	Uncertainty, Risk, Ambiguity, Prospect Theory, Model, Expected Utility, Subjective Probability, Probability, Information, Heuristics
2	Neural mechanism study of subjective value representation	Decision Making, Subjective Value, Reward, Prefrontal Cortex, Orbitofrontal Cortex, Anterior Cingulate, Risk Taking, Attention
3	Construction of a behavioral–neural cross-domain model for uncertainty decision-making	Neural Representation, Preference, Neuroeconomics, Behavior, Neurobiology

## Data Availability

No new data were created or analyzed in this study. Data sharing is not applicable to this article.
